# Microencapsulation of Metal-based Phase Change Material for High-temperature Thermal Energy Storage

**DOI:** 10.1038/srep09117

**Published:** 2015-03-13

**Authors:** Takahiro Nomura, Chunyu Zhu, Nan Sheng, Genki Saito, Tomohiro Akiyama

**Affiliations:** 1 Center for Advanced Research of Energy and Materials, Hokkaido University, Kita 13 Nishi 8, Kita-ku, Sapporo, 060-8628 Japan

## Abstract

Latent heat storage using alloys as phase change materials (PCMs) is an attractive option for high-temperature thermal energy storage. Encapsulation of these PCMs is essential for their successful use. However, so far, technology for producing microencapsulated PCMs (MEPCMs) that can be used above 500°C has not been established. Therefore, in this study, we developed Al-Si alloy microsphere MEPCMs covered by α-Al_2_O_3_ shells. The MEPCM was prepared in two steps: (1) the formation of an AlOOH shell on the PCM particles using a boehmite treatment, and (2) heat-oxidation treatment in an O_2_ atmosphere to form a stable α-Al_2_O_3_ shell. The MEPCM presented a melting point of 573°C and latent heat of 247 J g^−1^. The cycling performance showed good durability. These results indicated the possibility of using MEPCM at high temperatures. The MEPCM developed in this study has great promise in future energy and chemical processes, such as exergy recuperation and process intensification.

Advanced high-temperature thermal energy storage (HTTES) systems with high heat storage densities are required for advanced electrical generation systems. These include concentrated solar power (CSP) plants, future coal-fired power plants, and more. Thermal energy storage (TES) plays a vital role in CSP plants because it is subjected to the intermittencies of the source and to the shift between the electrical needs and the solar availability^1^. Innovations in CSP receivers, from trough to central receiver towers, provided an increase in the concentrated temperature, necessitating the development of HTTES up to 1000°C^1^. An advanced integrated coal gasification fuel cell combined cycle (A-IGFC) system, which includes the exergy recuperation process by recycling exhaust heat from fuel cells for coal steam gasification, is proposed for future coal-fired power plants^2^. Koda et al. reported that the gross electrical efficiencies of A-IGFCs could reach as high as 70–76% with a higher heating value^3^. Guan et al.^2^ stated that the system needs to be operated in high density and solid flux conditions (several 10^2^–10^3^ kg m^−2^ s^−1^) to sufficiently transfer heat to the endothermic pyrolysis and gasification reactions at about 1000°C^4^,^5^. Thus, a solid heat transfer medium with high heat density is needed. Sensible heat storage (SHS) technology using ceramics has already been established and applied as the conventional HTTES; however, its heat storage density is too low for the future technologies, necessitating HTTES with high heat storage density.

Under these circumstances, latent heat storage (LHS) has attracted considerable attention. LHS is based on the storage or release of latent heat when a phase change material (PCM) undergoes a phase transition from solid to solid, solid to liquid, or vice versa. LHS has three advantages: its latent heat gives it a large heat capacity compared to SHS, it can store and release heat when held constant at the phase transition temperature, and its reversible phase changing processes allow for repeated use^6^. These advantages allow LHS to be used as an advanced TES instead of SHS. LHS has been applied to the utilization of solar heat^7^,^8^ and industrial waste heat^9^,^10^, especially in the low-temperature range (<200°C).

Encapsulation of PCM is essential for its practical use because LHS mainly uses solid-liquid transformation and liquid PCM leakage must be prevented. Encapsulation also provides three more advantages for LHS: PCM capsules can provide sufficient surface area for heat transfer^11^; capsule walls act as barriers against harmful environmental reactions^11^; and they are structurally stable and easy to handle^11^just like solid particles.

Microencapsulation of PCM provides two additional great advantages to LHS. First, the microencapsulated PCM, with its high heat storage density, can be used as a mode of heat transfer and transport flux. The microencapsulated PCM flux can effectively recover, transport, and supply heat from a process to another process. Combinations of the PCM microcapsules with liquid^12^ or gas^13^,^14^ were reported. Second, microencapsulated PCM can be mixed with other materials and form multifunctional materials such as building materials and concretes^15^,^16^,^17^. These advantages have lead to many liquid-based microencapsulation methods only for low temperature PCMs, such as complex coacervation^18^, polymerization^19^,^20^,^21^, electrospinning^22^,^23^, and sol-gel methods^24^,^25^.

Many problems create difficulty in developing encapsulation technology for high temperature PCMs, such as molten salt and alloys, compared to low temperature PCMs. First, liquid metal and molten salt generally exhibit high chemical corrosion in the presence of shell materials like metals. Second, PCMs which transition from solid to liquid expand in volume. This is more serious for high temperature PCMs since a solid phase is always used as a starting material for encapsulation. Thermal stress caused by volume expansion must be addressed in capsule design. Finally, addressing the above problems necessitates thick shells, however, this drops the heat storage density.

Though macro-encapsulation (>1 mm) of high temperature PCMs has been reported, nothing has been established to simultaneously solve these three problems. G. Zhang et al.^26^ proposed encapsulating Cu-based PCM with a Cr-Ni bi-layer using a chromium periodic-barrel electroplating method and nickel barrel-plating method. The results showed that the capsule could endure charge-discharge thermal cycles without leakage, had excellent oxidation resistance, and exhibited stability between the inner Cu and Cr-Ni shell. However, the shell thickness was too thick and the heat storage density (50 J g^−1^) was as small as 20 wt% of its pure phase.

F. Piti et al.^27^ proposed a thermo-mechanical model describing the behavior of a free spherical PCM coated by SiC. Only under specific conditions can encapsulated PCM particles melt without cracking the coating shell. Low volumetric expansion PCM is necessary to decrease the pressure increase during melting. A. Mathur et al.^28^ indicated the necessity of a void inside the shell allowing for PCM volume expansion. They developed PCM (NaNO_3_) capsules with voids using a sacrificial polymer as the middle layer between the PCM pill and the shell material. However, their results do not refer the heat storage density of the capsules. In addition, microencapsulation of high-temperature PCMs, which have melting points greater than 500°C, has never been reported to the best of our knowledge.

Therefore, this study develops microencapsulated PCMs (MEPCM) for advanced high-temperature applications. There are three critical concepts to achieve the microencapsulation of high-temperature PCMs.

(1) ***Alloy use in PCMs***: Alloys generally expand less in volume during solid-liquid transition than molten salt. They have high thermal conductivity and provide high thermal response during heat storage and release. In this study, Al-25 wt%Si (melting point: 577°C, average diameter: 36.3 μm) was selected as the high-temperature PCM.

(2) ***Development of core-shell type capsules with alloy cores and oxide (Al_2_O_3_) shells***: Oxide resists corrosion caused by liquid metal and is easily formed by oxidation treatment of metallic particles.

(3) ***Microencapsulation of liquid PCMs***: Encapsulating the PCM in the liquid state, where the volume is largest, creates effective void for the solid to liquid phase expansion.

Based on these concepts, the MEPCM of Al-Si alloy was prepared using facile two-step methods: (1) the formation of an AlOOH shell on the PCM particles using a boehmite treatment, and (2) heat-oxidation treatment in an O_2_ atmosphere to form a stable α-Al_2_O_3_ shell. The developed MEPCM presented a melting point of 573°C, large latent heat of 247 J g^−1^ and perfect durability. These results indicated the possibility of using MEPCM at high temperatures.

## Results

### Characterization of the samples

**Figure 1:**
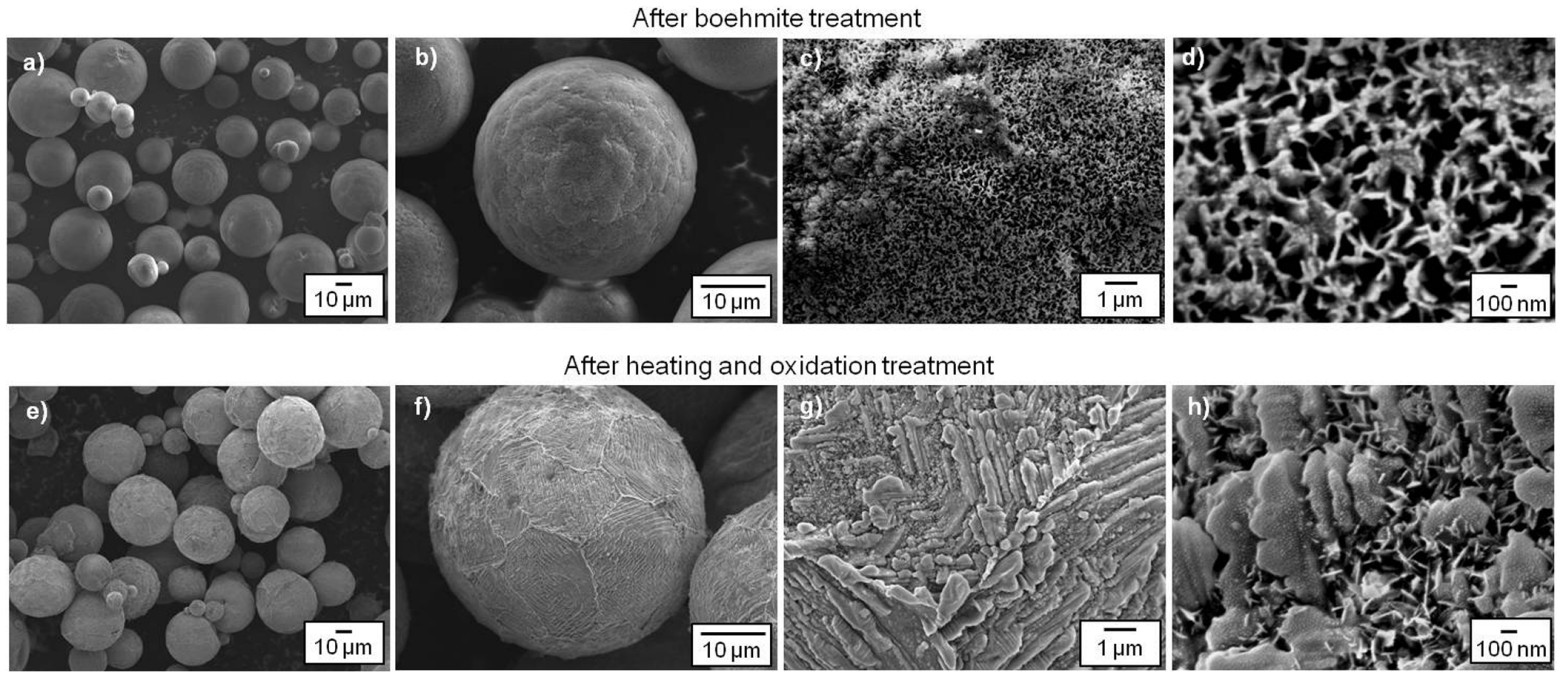
Scanning electron microscopy (SEM) images of the samples after (a–d) boehmite treatment and (e–h) heat-oxidation treatments.

Fig. 1 shows different magnifications of SEM images of the samples after boehmite (a–d) and heating-oxidation treatments (e–h). The spherical shape of Al-Si alloy was maintained after both boehmite and heat-oxidation treatments compared to the raw materials shown in Fig. S1 (Supplementary Information 1). However, a significant difference in the surface morphologies was found for those samples. The raw materials of Al-Si spheres possess a smooth surface, while the capsulated sample surfaces are rough and lumpy because of shell formation through recrystallization.

The boehmite-treated sample surface was carefully observed. Fig. 1(c) and (d) show its porous network consisting of interconnected fibrillar crystals (45–50 nm thick, 300–500 nm long). Fibrillar crystals form 300–500 nm pores. This architecture is called flower-like structure^29^. After heating and oxidation treatments, substantial differences in surface morphology are emphasized by the crystal growth of the flower-like shell. As shown in Fig. 1 (g) and (h), instead of fibrillar nanocrystals (300–500 nm long) after boehmite treatment, large lamellar crystals or diagonal pieces formed on the sphere surface after heating and oxidation treatments. The lamellar crystals were 10^−1^–1 μm thick and greater than 1 μm long. The sphere surfaces became dense after heating and oxidation treatment. Furthermore, no liquid PCM leakage was observed even after heating the sample to 930°C, higher than the Al-Si alloy melting point. This implied the successful formation of encapsulated PCM with a condensed and stable shell.

**Figure 2:**
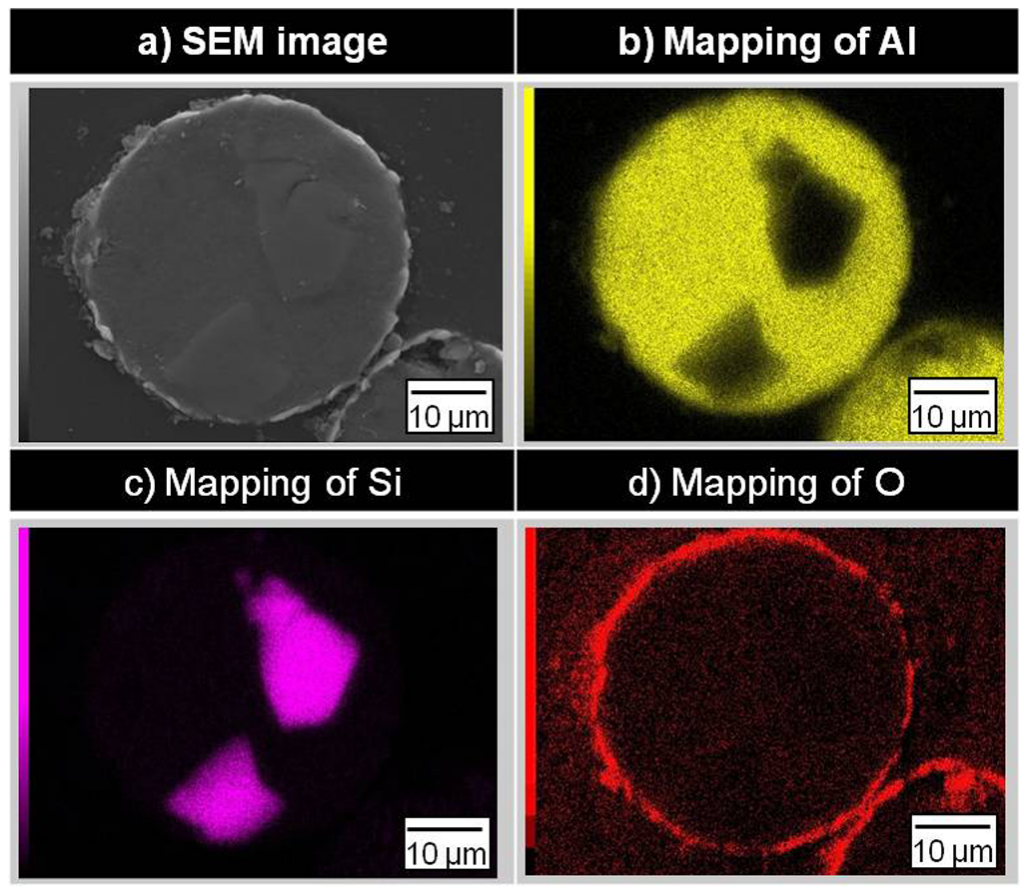
Energy dispersive spectroscopy (EDS) elemental mapping of the sample using the cross-section after heat-oxidation treatment.

Fig. 2 presents the elemental mapping of (b) Al, (c) Si, and (d) O on the cross-section of the samples after heat-oxidation treatment. Al and O were detected on the sphere surface producing a shell, O was not observed in the sphere core, and Si was not observed in the shell. This indicated that the capsule shell consisted solely of Al_2_O_3_.

**Figure 3:**
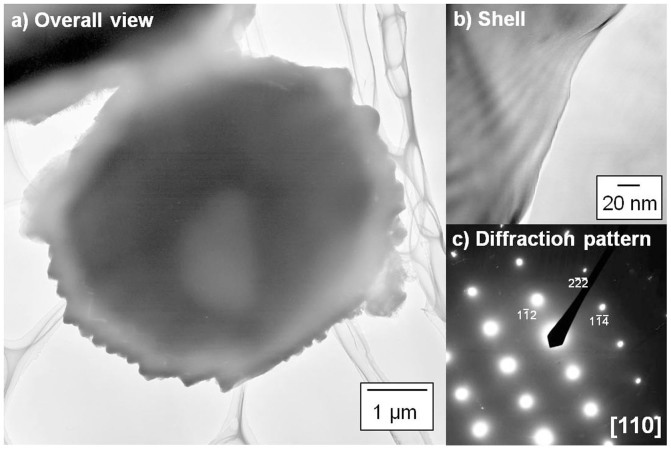
Transmission electron microscopy (TEM) images of (a, b) the microencapsulated phase change material (MEPCM), and (c) the diffraction pattern taken from the shell.

Fig. 3 presents TEM images of the MEPCM and the selected area diffraction pattern taken from its shell. The TEM images show that the shell was structurally dense with a rough surface. The shell diffraction pattern can be indexed to [110] of α-Al_2_O_3_, indicating the formation of a dense and stable α-Al_2_O_3_ shell.

**Figure 4:**
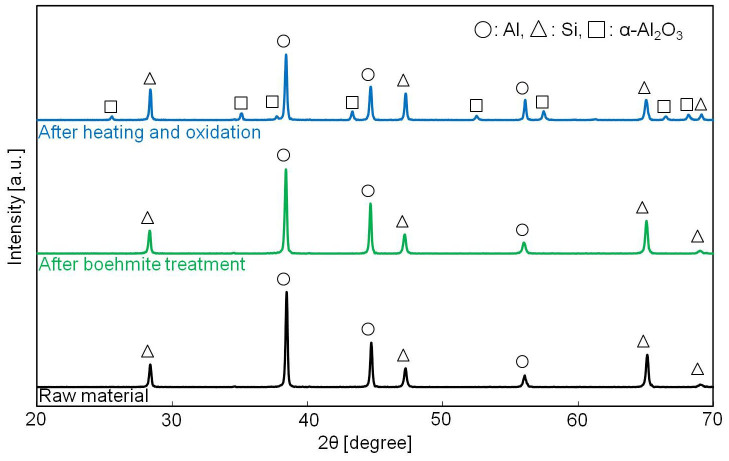
X-ray diffraction (XRD) patterns for the raw material, the sample after boehmite treatment, and the sample after heating and oxidation.

Fig. 4 shows XRD patterns of the raw material, the sample after boehmite treatment, and the sample after heating and oxidation treatment. The XRD peaks of the raw material and the sample after boehmite treatment can be indexed to those of Al and Si, although after boehmite treatment the sample should be covered by an AlOOH shell. This indicated that the as-prepared boehmite shell was amorphous. On the other hand, α-Al_2_O_3_ was detected in the sample after heat-oxidation treatment. α-Al_2_O_3_ is thermodynamically the most stable form of Al_2_O_3_ and has the best mechanical properties and corrosion resistance of the forms of Al_2_O_3_, making it an ideal shell. The MEPCM composition was determined using XRD quantitative analysis to be Al/Si/Al_2_O_3_ = 45%/30%/25% in weight ratio. Since Al_2_O_3_ only exists in the MEPCM shell, the core composition was calculated to be Al/Si = 60%/40% in weight ratio.

The measurement of particle size distributions (Fig. S2 in Supplementary information 2) showed that the average diameters of the raw material, *D_ave,__raw_* was 36.3 μm, and that of the samples after treatment, *D_ave,MEPCM_* was 40.7 μm. Shell thickness, *d_shell_*, was estimated by the following equation: 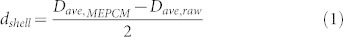
As a result, *d_shell_* of MEPCM was 2.2 μm.

**Figure 5:**
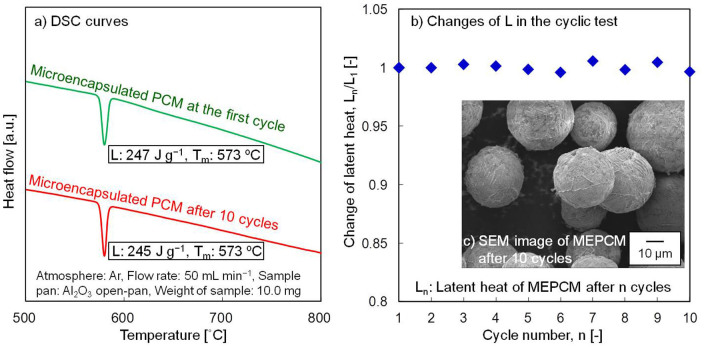
(a) DSC curves of MEPCM at the first cycle and MEPCM after 10 cycles of repeated melting and freezing in air, (b) the changes in the latent heat of MEPCM in the cyclic test, and (c) SEM image of MEPCM after 10 cycles of repeated melting and freezing.

Fig. 5 a) shows the DSC curves of MEPCM at the first cycle and after 10 cycles of repeated melting and freezing in air. The phase change temperature of the as-prepared MEPCM was 573°C, which was close to that of non-encapsulated PCM as shown in Fig. S3 (Supplementary information 3). The latent heat of the MEPCM was 247 J g^−1^, which is about 57% that of the raw material. Considering both the change in composition from Al-25 wt%Si to Al-40 wt%Si of the PCM core and the formation of Al_2_O_3_ shell, which occupied 25 wt% of the MEPCM, this value is close to the theoretical one. It should be emphasized that our MEPCM latent heat is large among reported values of encapsulated PCMs. For example, the latent heat of macro-encapsulated Cu, produced using an electroplating method, was about 50 J g^−1^. This value is as small as 20 wt% of its pure phase^26^. Fig. 5 b) shows the changes in the L of MEPCM in the cyclic test. As shown in Fig. 5 b), even after 10 cycles, the phase change temperature and the latent heat of the MEPCM were almost consistent with those at the first cycle. Fig. 5 c) shows the SEM image of MEPCM after 10 cycles of repeated melting and freezing. We can clearly observe that the as-prepared MEPCM has kept its original structure without leakage.

## Discussion

This study achieved Al-Si alloy microsphere MEPCMs covered by α-Al_2_O_3_ shells, with large latent heat and perfect durability. The discussion will mainly focus on the formation mechanism of the final core-shell structure from chemical and mechanical viewpoints.

There are two reasons for α-Al_2_O_3_ formation on the surface. First, the boehmite film shown in Fig. 1 c)–d) transformed to α-Al_2_O_3_ during heat treatment because it was the aluminum oxide precursor. Boehmite-treated film is reported to be a duplex structure consisting of a dense inner layer and a porous fibrillar outer layer^30^, and our boehmite film exhibited this structure since its flower-like structure is identical with the porous and fibrillar outer layer of a boehmite-treated Al plate^29^. This indicated that the dense inner layer of boehmite transformed to dense α-Al_2_O_3_ shells as shown in Fig. 1 g)–h) and Fig. 3. Second, the selective oxidation of Al in the Al-Si alloy occurred because Al has a stronger oxygen affinity than Si (Fig. S4 in Supplementary information 4). The shell consisting of a single aluminum oxide composition showed high shell strength. A shell consisting of both silicon and aluminum oxides would exhibit decreased shell strength because of the different thermal expansion coefficients.

The MEPCM composition was determined using XRD quantitative analysis to be Al/Si = 60%/40% in weight ratio, showing an increase in the amount of Si compared to the raw material (Al/Si = 75%/25%). This is because Al was selectively consumed during boehmite and heat-oxidation treatments to transfer to the Al_2_O_3_ shell.

**Figure 6:**
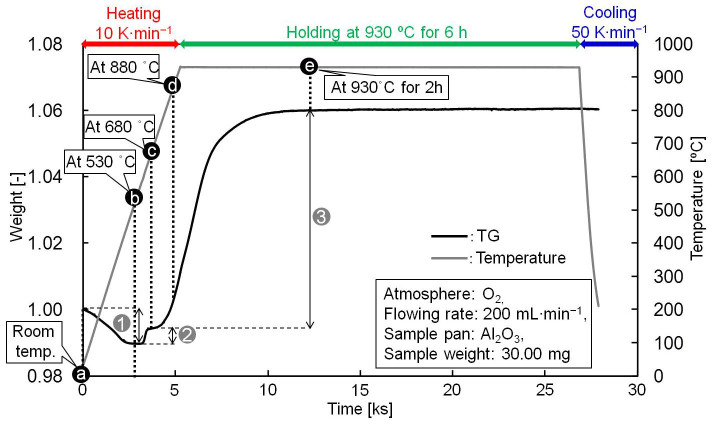
Thermogravimetric (TG) curve of the boehmite-treated sample during heat-oxidation treatment. Note that labels (a–d) correspond to those in Fig. 7, in which the samples was taken out for SEM observation.

To investigate the formation mechanism of the final core-shell structure from mechanical viewpoints, the boehmite-treated sample was used for TG analysis. The sample was heated to different temperatures and its morphology was observed. Fig. 6 illustrates the TG curve from heating the boehmite-treated sample at a rate of 10 K min^−1^ to 930°C and staying at this temperature for 6 h under an O_2_ flow.

The TG curve showed three obvious weight jumps. The first occurred from room temperature to 530°C near the Al-Si eutectic temperature (the transition temperature, *T_m_*, from solid phase to liquid phase: 577°C), showing a 1.0% weight drop. This was due to the dehydration of AlOOH:
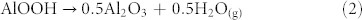


The second weight change, a sudden increase of 0.5%, was observed in the 530–650°C range, which is slightly higher than the PCM transition temperature. The third change occurred around 705°C and was a 6.6% increase. The sample weight stabilized after having been 930°C for 40 min.

**Figure 7:**
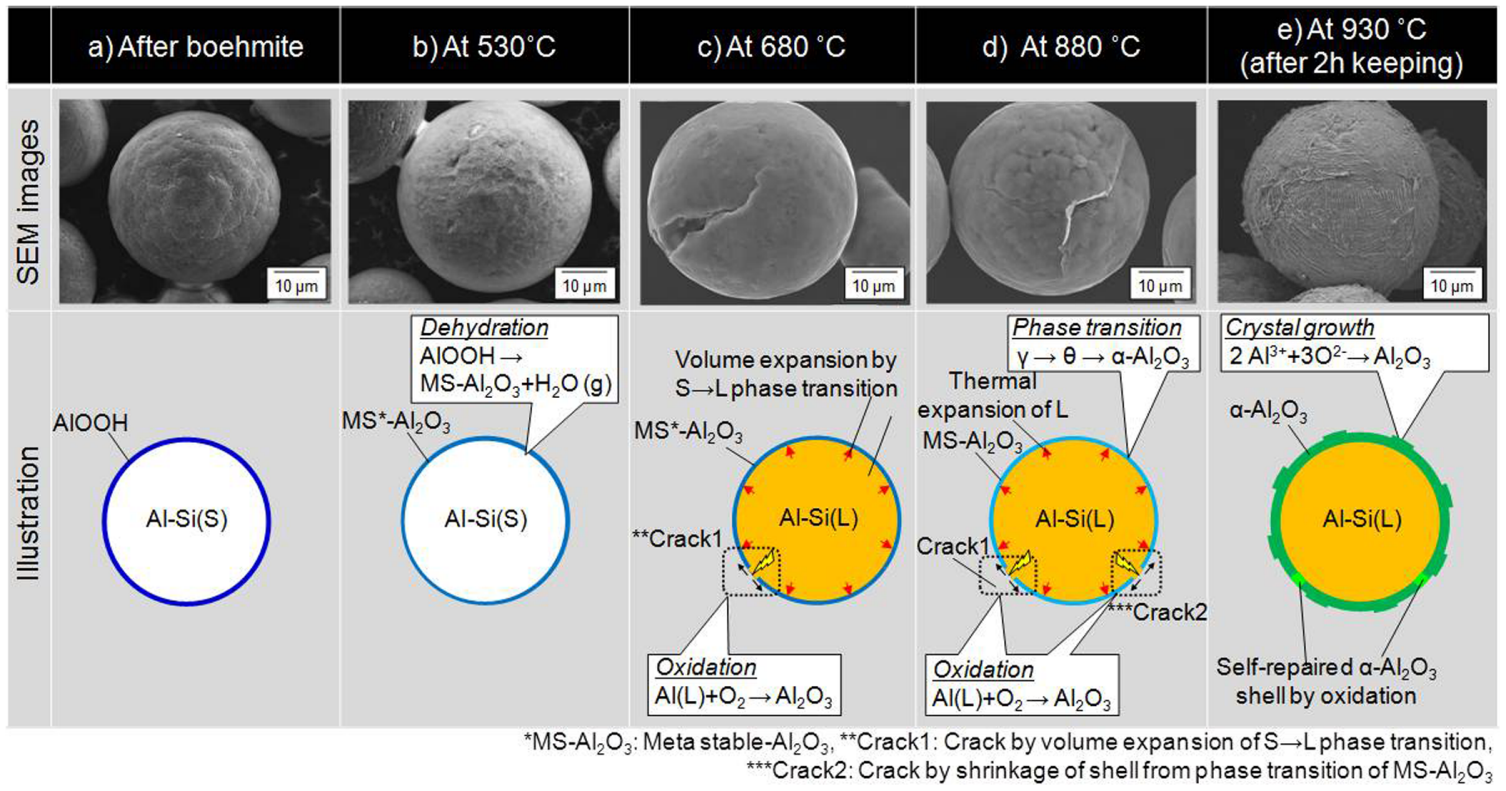
SEM images and schematic diagram of the sample stopped at different stages during heat-oxidation treatment. (a–e) correspond to the those pointed in Fig. 6.

Changes in morphology during heat-oxidation treatment were investigated by examining five samples obtained by stopping the heating at different stages based on characteristics of the TG curve. The samples were the (a) boehmite-treated raw material, (b) sample heated to 530°C, (c) sample heated to 680°C, (d) the sample heated to 880°C, and (e) sample heated to and held at 930°C for 2 h. Fig. 7 presents their corresponding SEM images. There was no significant difference in the morphologies of samples (a) and (b), although AlOOH underwent dehydration at this temperature. However, sample (c) showed a crack in the shell. This crack was caused by rapid volume expansion of the PCM core due to the solid to liquid phase transition at a temperature higher than *T_m_*. As a result, 530–650°C exhibited a sudden weight increase. This was due to the quick oxidation of Al, now exposed to oxygen near the crack:
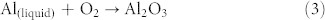


The weight increase seemed to stop over the 650–705°C range. This indicated the formation of a temporary stable oxidation film on the interfacial surface of the exposed liquid Al that prevented the leakage and oxidation of interior liquid Al. Furthermore, the partial transformation of solid Si to liquid Si in this range, according to its phase diagram as shown in Fig. S5 (Supplementary information 5), and significantly suppressed the total PCM volume expansion because Si shrunk by as much as 9.5%^31^ during its phase change.

A marked weight increase continued above 705°C. Fig. 7 (d), the sample stopped at 880°C, shows the morphological observations of this phenomenon. The crack in the shell seemed to have self-repaired with the large weight increase. In this stage, the following phenomenon seemed to occur: cracks generated on the shell due to thermal expansion of the shell material caused by volume expansion of the PCM core; interior Al was exposed to O near the cracks and quickly oxidized to form Al_2_O_3_ film; repeatedly generated new cracks, caused by heating, were quickly repaired by rapid Al oxidation. Oxidation due to the diffusion of oxygen into the spheres (Equation 4) accelerated as the temperature increased.
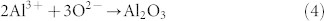


Finally, when heated to 930°C for several minutes, the weight stabilized. Fig. 7 (e) shows the SEM image of the sample heated to and held at 930°C for 2 h. A dense shell with coarse lamellar crystals on its surface formed uniformly. The oxide protective layer shell was calculated to be about 2.2 μm thick, which is enough to prevent interior liquid Al oxidation. Furthermore, crack generation stopped with the cessation of interior PCM volume expansion and shell material thermal expansion with temperature stabilization. An illustration of the formation mechanism of the core-shell structure is also presented in Fig. 7.

It is important that this research to successfully produces microencapsulated metallic PCM by combining boehmite treatment lower than *T_m_* and heat-oxidation treatment above *T_m_*. If no boehmite shell precursor formed, the Al-Si particles cannot keep its original shape because it transitions to liquid when heated above *T_m_*. Furthermore, the self-repairing function of the pre-coated PCM during oxidation treatment is another key to its success, with which a stable oxide layer could be formed.

F. Piti et al.^27^ reported a thermo-mechanical model for a ceramic encapsulated PCM sphere with a NaNO_3_ core and SiC shell. Their results indicated that the encapsulated PCM particles could only melt without cracking the coating shell under specific conditions because a high thermal stress occurs during the solid to liquid phase transition^27^. G. Zhang et al.^26^ proposed an encapsulation method for copper-based PCMs with chromium-nickel bi-layers using electroplating. The capsulated PCM showed endurance useful for thermal storage/release after long cycles without leakage, however, the shell of the capsulated PCM, several millimeters, was too thick, significantly reducing the latent heat of the designed PCM.

Unlike the above encapsulation methods, ours produced the final stable oxide shells at high temperature after the PCM had fully expanded in its liquid form. Therefore, void is created when the PCM cools. In other words, when the as-prepared MEPCM is used in a lower temperature area than its treatment temperature, enough voids exist in the capsule to compensate for its volume change during heating. Therefore, the as-prepared MEPCM has a high thermo-mechanical characteristic.

The necessity for voids inside the encapsulated PCM to compensate for the volume expansion during melting has been pointed out by A. Mathur et al.^28^. This has been used for producing a variety of low-temperature PCMs^17^,^18^,^19^,^20^,^21^,^22^,^23^,^24^,^25^, but this study is the first attempt at producing high-temperature metallic MEPCM.

**Figure 8:**
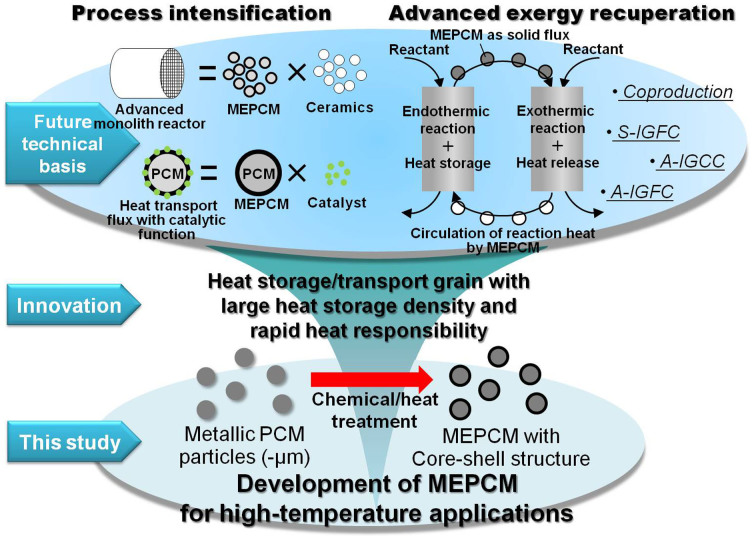
Expected MEPCM applications. (A-IGFC: Advanced integrated coal gasification fuel-cell combined cycle; A-IGCC: Advance integrated coal gasification combined cycle; S-IGFC: Super integrated coal gasification fuel-cell combined cycle).

In summary, this paper showed the development of an MEPCM consisting of an Al-40 wt%Si alloy core/PCM and an α-Al_2_O_3_ shell. The MEPCM showed characteristics such as high heat storage density, high thermal responsibility, and high endurance for heat storage or transport media, for high temperature applications. The developed MEPCM have great promise in future energy and chemical processes, such as exergy recuperation^5^ and process intensification^32^. Fig. 8 presents the expected applications of MEPCMs. Since the high-temperature MEPCM can be used in the form of a solid-flux with high heat storage density and high heat transfer speed, it could be applied to advanced chemical processes which transport heat generated by exothermic reactions to endothermic reactions, such as A-IGFC or the co-production of chemicals. Additionally, referring to the similar system of low-temperature PCM, there was a possibility to develop an advanced monolith reactor with high heat storage density and homeothermic function using a ceramic-MEPCM composite. This material will be important in the field of process intensification^32^.

## Methods

### Materials

Micro-spherical particles of Al-25 wt%Si, which is a hypereutectic Al-Si alloy, were used as raw materials. Spinning disk atomization was used to produce particles 36.3 μm in average diameter. Fig. S5 (Supplementary information 5) shows the phase diagram of the Al-Si binary alloy. Fig. S1 (Supplementary information 1) presents scanning electron microscope (SEM) images of the micro-spherical particles.

### Preparation of microencapsulated PCM

The MEPCM was prepared in two steps as shown in Fig. S6. (Supplementary information 6). First, boehmite treatment for AlOOH preparation shell by boiling the microspheres in distilled water for 3 h. Then the sample was filtrated and dried at 105°C over night. Second, the microspheres are subjected to heat-oxidation treatments, and a thermobalance-apparatus (METTLER TOLEDO TG-DSC-1) was used to precisely control the temperature and atmosphere. The boehmite-treated sample was heated from room temperature to 930°C at a rate of 10 K min^−1^, kept at this temperature for 6 h, and finally cooled to room temperature at a rate of 50 K min^−1^. Treatment was performed using 30 mg of specimens in an Al_2_O_3_ crucible at an O_2_ (Purity: 99.5%) flow rate of 200 ml min^−1^. To investigate the formation mechanism of the core-shell material, samples were stopped at different treating stages.

### Material characterization

Sample morphology was evaluated by SEM (JEOL, JSM-7400F) and transmission electron microscopy (TEM, JEOL JEM-2010F). Sample element distribution after heat-oxidation treatments were observed by energy dispersive spectroscopy (EDS, JEOL, JSM-7001FA). Phase compositions were characterized by powder X-ray diffraction (XRD, Rigaku Miniflex, Cu Ka). The particle size distribution was measured by a particle size distribution analyzer (HORIBA, LA-950) based on Mie scattering theory. The Melting point, freezing point, and latent heat were measured using a combined thermogravimetry (TG) and differential scanning calorimetry (DSC) analyzer (TGA/DSC1, METTLER TOLEDO). Samples were subjected to a heating rate and cooling of 2 K min^−1^ in an Ar atmosphere.

### Cyclic test

Cyclic tests were performed on the microencapsulated spheres to evaluate their durability by repeating the melting and freezing processes up to 10 cycles. In this measurement, 30 mg of specimen was repeatedly heated to 800°C and cooled to 500°C at a rate of 50 K min^−1^ using an Al_2_O_3_ crucible in an air flow of 200 ml min^−1^.

## Author Contributions

T.N. conceived the idea, conducted experiments, and wrote the manuscript. C.Z. and N.S. helped with experimental work and data analysis, reviewed and commented on the manuscript. G.S. prepared Fig. 3. T.A. supervised the project.

## Supplementary Material

Supplementary Information Supplementary information

## References

[b1] Guillot S. *et al.* Corrosion effects between molten salts and thermal storage material for concentrated solar power plants. Appl. Energ. 94, 174–181 (2012).

[b2] Guan G. *et al.* High-density circulating fluidized bed gasifier for advanced IGCC/IGFC—Advantages and challenges. Particuology 8, 602–606 (2010).

[b3] Koda E. *et al.* Conceptual design of an A-IGFC *(In Japanese)*. Proceedings of the Japan Society of Chemical Engineers 37th autumn meeting, Okayama, Japan. Tokyo: Japan Society of Chemical Engineers. (September 17 2005).

[b4] Bi X. T. & Liu X. High density and high solids flux CFB risers for steam gasification of solids fuels. Fuel Process.Tech. 91, 915–920 (2010).

[b5] Tsutsumi A. Advanced IGCC/IGFC using exergy recuperation technology *(in Japanese)*. Clean Coal Technol. J. 11, 17–22 (2004).

[b6] Nomura T., Tsubota M., Oya T., Okinaka N. & Akiyama T. Heat storage in direct-contact heat exchanger with phase change material. Appl. Therm. Eng. 50, 26–34 (2013).

[b7] Gil A. *et al.* State of the art on high temperature thermal energy storage for power generation. Part 1—Concepts, materials and modellization. Renew. Sustain. Energ. Rev. 14, 31–55 (2010).

[b8] Laing D., Bauer T., Breidenbach N., Hachmann B. & Johnson M. Development of high temperature phase-change-material storages. Appl. Energ. 109, 497–504 (2013).

[b9] Kaizawa A. *et al.* Thermal and flow behaviors in heat transportation container using phase change material. Energ. Convers. Manag. 49, 698–706 (2008).

[b10] Nomura T., Okinaka N. & Akiyama T. Waste heat transportation system, using phase change material (PCM) from steelworks to chemical plant. Resour. Conservat. Recycl. 54, 1000–1006 (2010).

[b11] Regin A. F., Solanki S. & Saini J. Heat transfer characteristics of thermal energy storage system using PCM capsules: A review. Renew. Sustain. Energ. Rev. 12, 2438–2458 (2008).

[b12] Yamagishi Y., Takeuchi H., Pyatenko A. T. & Kayukawa N. Characteristics of microencapsulated PCM slurry as a heat-transfer fluid. AIChE J. 45, 696–707 (1999).

[b13] Izquierdo-Barrientos M., Sobrino C. & Almendros-Ibáñez J. Thermal energy storage in a fluidized bed of PCM. Chem. Eng. J. 230, 573–583 (2013).

[b14] Pitié F., Zhao C., Baeyens J., Degrève J. & Zhang H. Circulating fluidized bed heat recovery/storage and its potential to use coated phase-change-material (PCM) particles. Appl. Energ. 109, 505–513 (2013).

[b15] Cabeza L., Castell A., Barreneche C., De Gracia A. & Fernández A. Materials used as PCM in thermal energy storage in buildings: a review. Renew. Sustain. Energ. Rev. 15, 1675–1695 (2011).

[b16] Khudhair A. M. & Farid M. M. A review on energy conservation in building applications with thermal storage by latent heat using phase change materials. Energ. Convers. Manag. 45, 263–275 (2004).

[b17] Tyagi V., Kaushik S., Tyagi S. & Akiyama T. Development of phase change materials based microencapsulated technology for buildings: a review. Renew. Sustain. Energ. Rev. 15, 1373–1391 (2011).

[b18] Hawlader M., Uddin M. & Khin M. M. Microencapsulated PCM thermal-energy storage system. Appl. Energ. 74, 195–202 (2003).

[b19] Alkan C., Sarı A., Karaipekli A. & Uzun O. Preparation, characterization, and thermal properties of microencapsulated phase change material for thermal energy storage. Sol. Energ. Mater. Sol. Cell. 93, 143–147 (2009).

[b20] Chaiyasat P., Islam M. Z. & Chaiyasat A. Preparation of poly(divinylbenzene) microencapsulated octadecane by microsuspension polymerization: oil droplets generated by phase inversion emulsification. RSC Adv. 3, 10202–10207 (2013).

[b21] Sarı A., Alkan C., Karaipekli A. & Uzun O. Microencapsulated n- octacosane as phase change material for thermal energy storage. Sol. Energ. 83, 1757–1763 (2009).

[b22] Hu W. & Yu X. Encapsulation of bio-based PCM with coaxial electrospun ultrafine fibers. RSC Adv. 2, 5580–5584 (2012).

[b23] McCann J. T., Marquez M. & Xia Y. Melt coaxial electrospinning: a versatile method for the encapsulation of solid materials and fabrication of phase change nanofibers. Nano lett. 6, 2868–2872 (2006). 17163721 10.1021/nl0620839

[b24] Ciriminna R., Sciortino M., Alonzo G., Schrijver A. D. & Pagliaro M. From molecules to systems: sol − gel microencapsulation in silica-based materials. Chem. Rev. 111, 765–789 (2010). 20726523 10.1021/cr100161x

[b25] Zhang H., Wang X. & Wu D. Silica encapsulation of n-octadecane via sol–gel process: A novel microencapsulated phase-change material with enhanced thermal conductivity and performance. J. Colloid Interface Sci. 343, 246–255 (2010). 20035943 10.1016/j.jcis.2009.11.036

[b26] Zhang G. *et al.* Encapsulation of copper-based phase change materials for high temperature thermal energy storage. Sol. Energ. Mater. Sol. Cell. 128, 131–137 (2014).

[b27] Pitie F., Zhao C. Y. & Caceres G. Thermo-mechanical analysis of ceramic encapsulated phase-change-material (PCM) particles. Energ. Environ. Sci. 4, 2117–2124 (2011).

[b28] Mathur A., Kasetty R., Oxley J., Mendez J. & Nithyanandam K. Using encapsulated phase change salts for concentrated solar power plant. Energy Procedia 49, 908–915 (2013).

[b29] Tadanaga K., Katata N. & Minami T. Formation Process of Super-Water-Repellent Al_2_O_3_ Coating Films with High Transparency by the Sol–Gel Method. J. Am. Ceram. Soc. 80, 3213–3216 (1997).

[b30] Kudo T. & Alwitt R. S. Cross-sections of hydrous and composite aluminum oxide films. Electrochim. Acta 23, 341–345 (1978).

[b31] Japan Society of Thermophysical Properties, Thermophysical properties handbook (*in Japanese)*, (eds. Nagashima, A. *et al.*) Ch 5, 103 (Yokendou, 2008).

[b32] Stankiewicz A. I. & Moulijn J. A. Process intensification: transforming chemical engineering. Chem. Eng. Progr. 96, 22–34 (2000).

