# Supplementary material for: Microencapsulation of Metal-based Phase Change Material for High-temperature Thermal Energy Storage
Source: Sci Rep. 2015 Mar 13;5:9117. doi: 10.1038/srep09117 (PMC4357867; doi:10.1038/srep09117)
Supplement: Supplementary Information [file srep09117-s1.doc]

Supplementary Information

Title

**Microencapsulation of Metal-based Phase Change Material**

**for High-temperature Thermal Energy Storage**

***Takahiro Nomura,^a*^ Chunyu Zhu,^a^ Nan Sheng,^a^ Genki Saito,^a^ and Tomohiro Akiyama^a^***

*^a^Center for Advanced Research of Energy and Materials, Hokkaido University, Kita 13 Nishi 8, Kita-ku, Sapporo, 060-8628 Japan*

**Corresponding author.*

*Tel.: +81 11 706 6842; fax: +81 11 706 6849*

*E-mail address:* [*nms-tropy@eng.hokudai.ac.jp*](mailto:nms-tropy@eng.hokudai.ac.jp) *(T.Nomura)*

Keywords: Phase change material, Microencapsulation, Latent heat Storage, Thermal Energy Storage, Aluminum Oxide

**Supplementary information 1**

**Fig. S1** presents scanning electron microscope (SEM) images of Al-25wt%Si micro-spherical particles with average diameters of 36.3 μm produced using the spinning disk atomization method.

**Figure S1. Scanning electron microscopy (SEM) images of the a) raw material and b) Al-25wt%Si, which were produced by spinning disk atomization. It was observed that most particles were spherical.**

**Supplementary information 2**

**Fig. S2** illustrates the particle size distributions of the raw material, sample after boehmite treatment, and sample after heat-oxidation treatment. D_ave_ means average pore diameter.

**Figure S2. Particle size distributions of the raw material, sample after boehmite treatment, and sample**

**after heating and oxidation.**

**Supplementary information 3**

**Fig. S3** shows the DSC curve of non-encapsulated PCM, Al-25wt%Si. The result indicated that the phase change temperature of the raw material was 577 °C and the latent heat was 432 J∙g^−1^.

**Figure S3. DSC curve of non-encapsulated PCM, Al-25wt%Si.**

**Supplementary information 4**

**Fig. S4** compares the oxygen affinities of Al and Si. Here R is the gas constant, T is temperature, PO_2_ is the oxygen partial pressure, and ΔG˚ is the standard free energy change. RTln(PO_2_) is equal to ΔG˚ in oxide formation reactions. Therefore, a lower RTln(PO_2_) value signifies a stronger oxygen affinity. Al has a stronger oxygen affinity than Si over the whole temperature range as a result.

**Figure S4. Comparison of the oxygen affinities of Al and Si. RTln(PO_2_) is equal to the standard free energy change, ΔG˚, of oxide formation reactions. (R: Gas constant, T: Temperature, PO_2_: Partial pressure of oxygen)**

**Supplementary information 5**

**Fig. S5** shows the binary phase diagram of the Al-Si alloy calculated using Factsage 6.4. Al-25wt%Si is a hypereutectic composition, meaning Si is composed over a eutectic mixture.

**Figure S5. Binary phase diagram of Al-Si alloy calculated using Factsage 6.4.**

**Supplementary information 6**

**Fig. S6** shows the schematic diagram for the preparation of MEPCM.

**Figure S6. Experimental procedure to prepare MEPCMs. The MEPCMs were prepared by two steps: (1) AlOOH shell preparation by boehmite treatment, (2) Al_2_O_3_ shell preparation by heat and oxidation treatment.**
